# Controlling time-related biases in ECPR observational studies

**DOI:** 10.1186/s13054-026-06424-9

**Published:** 2026-11-06

**Authors:** Tal Soumagnac, Lucie Fanet, Alain Cariou, Wulfran Bougouin

**Affiliations:** 1 SAMU de Paris and Intensive Care Unit, Necker Hospital, 149 rue de Sèvres, Paris, 75015 France; 2 Medical Intensive Care Unit, Cochin Hospital, 27 rue du Faubourg Saint-Jacques, Paris, 75014 France; 3 Université Paris Cité, 12 rue de l’école de Médecine, Paris, France; 4 Université Paris Cité, Inserm, PARCC, Rue Leblanc, Paris, 75015 France; 5 Medical-Surgical Intensive Care Unit, Jacques Cartier Hospital, 6 Avenue du Noyer Lambert, Massy, 91300 France

Extracorporeal cardiopulmonary resuscitation (ECPR) has emerged as a second-line treatment for refractory out-of-hospital cardiac arrest (OHCA) when applied to selected patients and performed by trained teams within equipped systems of care [1]. However, interpreting observational studies that compare ECPR with conventional cardiopulmonary resuscitation (CCPR) remains challenging because treatment allocation is intrinsically time-dependent. Failing to account for temporal biases risks introducing false positive or false negative associations, yet statistical methods to control for them are limited.

As previously described, when evaluating patients undergoing CCPR, “resuscitation time bias” arises when some patients achieve an early return of spontaneous circulation (ROSC) and never reach the point at which they would be eligible for the exposure of interest (ECPR) [2]. These early survivors achieve ROSC after a short low-flow duration (defined as the time from the start of cardiopulmonary resuscitation to the ROSC) and, by definition, would never have been candidates for ECPR. Because these patients exhibit distinct biological phenotypes and shorter ischemia-reperfusion injuries, they seem less comparable to patients with prolonged OHCA, such as those reported in ECPR literature (median low-flow duration of 74 min in the INCEPTION trial [3], 59 min in the ARREST trial [4], and 58 min in Belohlavek et al. [5]).

An opposing form of “immortal time bias” affects ECPR patients in observational studies [2]. ECPR exposure cannot be established instantaneously and requires a logistical interval, reported as 20 min in the INCEPTION trial [3], 12 min in the ARREST trial [4], and 12 min in Belohlavek et al. [5]. Individuals who ultimately receive ECPR represent a highly selected, resilient cohort whose survival status is suspended following the clinical decision to be exposed to ECPR. This state persists during transport logistics, cannulation, and up to pump initiation. Such guaranteed survival is artificially built into the ECPR group definition, reflecting management strategy rather than the natural history of OHCA, whereas survival status remains variable over time in the CCPR group. Unlike randomized controlled trials that explicitly allocate patients at randomization, observational studies cannot capture this decision moment in registry data. Thus, the survival time accumulated while resuscitation efforts are prolonged awaiting ECPR is often misallocated to the CCPR group, creating design-related confounding.

Finally, the decision to initiate ECPR is itself complex and multifactorial. Outside the strict framework of a randomized controlled trial, this decision is made at different stages by different people, involving the on-scene physician, the receiving emergency department team, or the regional emergency dispatch center. The precise moment at which CCPR transitions into a bridge-to-ECPR strategy remains difficult to define retrospectively, particularly regarding decisions to withhold ECPR in the CCPR group, which are rarely documented in registries.

Consequently, observational studies of ECPR are particularly vulnerable to time-related biases. Early ROSC among CCPR patients biases estimates against ECPR, whereas immortal time preceding cannulation biases them in its favor. Moreover, as CCPR is frequently terminated after 20–30 min of advanced life support [6], few clinically comparable CCPR patients remain beyond this point (Fig. 1), with the probability of ROSC falling below 20% by 40 min [7]. Analyses that do not align time zero or adequately account for low-flow duration should therefore be interpreted with caution. Time dependent, adjusted multivariable regression models, and propensity score-based methods may partially mitigate these issues, but they are only effective if matching before exposure is adequate regarding low-flow duration and OHCA clinical severity, a requirement that results in a restricted overlap.

Landmark analyses can be considered to control for resuscitation time bias by excluding patients before a specific clinical milestone (e.g., 20–30 min of CCPR) [8]. However, implementing a temporal landmark removes patients whose CCPR was terminated before the chosen time point, and might result in a lack of patients for later comparison. Conversely, shortening this time criterion reintroduces CCPR survivors who achieved early ROSC and were never eligible for ECPR, thereby reintroducing “resuscitation time bias” [2].

Target trial emulation has also been advocated as a methodology to avoid bias in observational research [9]. To appropriately emulate randomization, patients should theoretically have a non-zero probability of receiving either ECPR or continued CCPR at each relevant time point (sequential target trial emulation; whereas selecting a single time-point approximates a well-defined landmark analysis). Nevertheless, at prolonged low-flow durations, this assumption becomes clinically and ethically challenging because CCPR is no longer an equivalent therapeutic alternative to ECPR. Simulating prolonged CCPR in patients who would otherwise undergo ECPR or cessation of care generates counterfactual scenarios with limited biological and ethical plausibility, and a loss of experimental positivity.

Quasi-experimental designs include the use of an instrumental variable exploiting exogenous variation in the likelihood of receiving ECPR. However, identifying a valid and sufficiently relevant instrument is challenging. Methods previously applied in other cardiac conditions, such as differential distance to specialized centers [10], may be difficult to apply in OHCA, as transport time and distance are themselves directly associated with mortality. Interrupted time-series analyses provide an alternative by comparing outcomes before and after ECPR program implementation. Beyond requiring enough patients to capture an relatively infrequent event under current ECPR eligibility criteria, such analyses must also account for concurrent improvements in overall OHCA care that could confound the effect of ECPR program implementation over time.

**Fig. 1:**
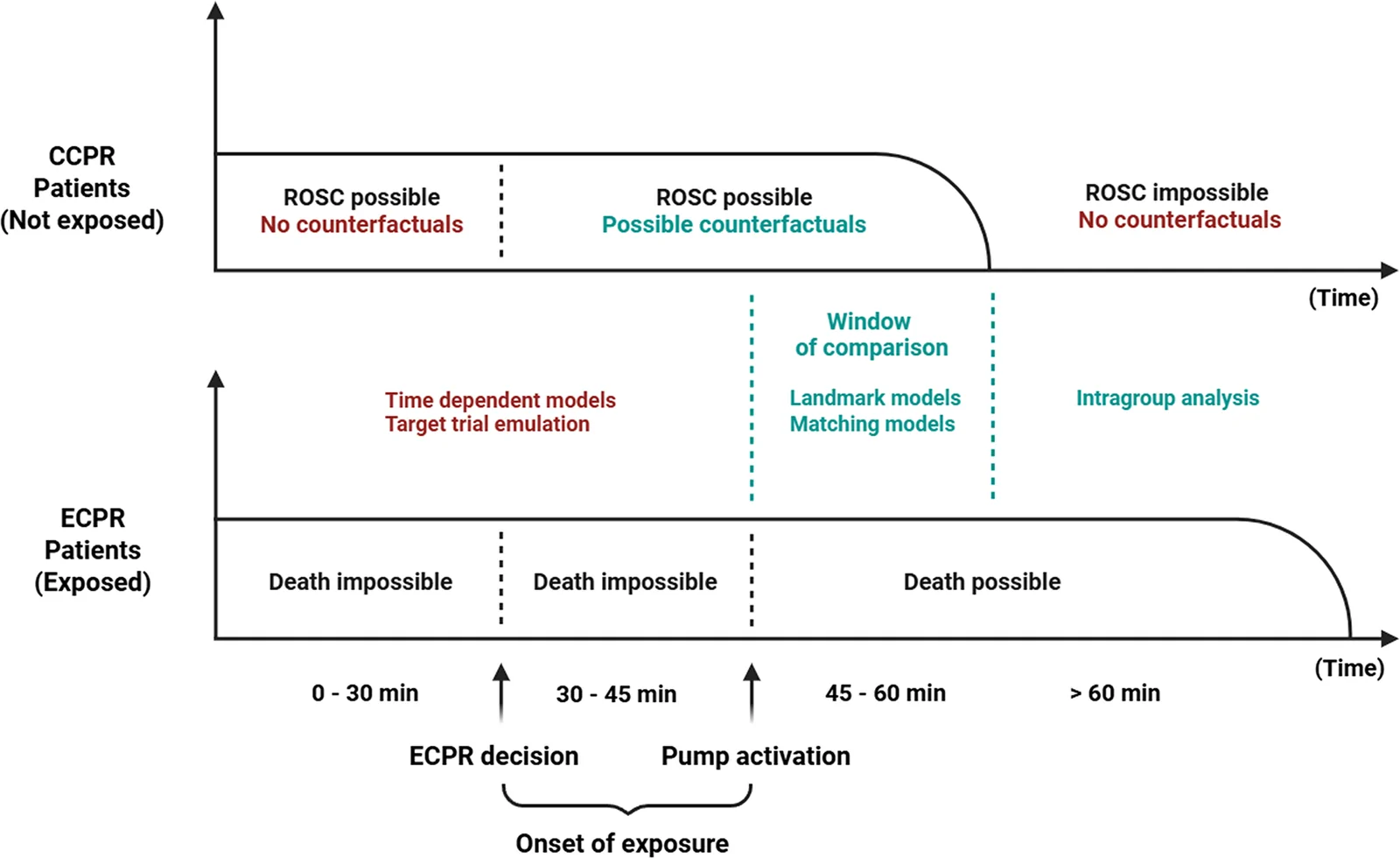
Time-Related Biases in ECPR Observational Studies. Legend: ECPR: extracorporeal cardiopulmonary resuscitation; ROSC: return of spontaneous circulation. This figure is adapted from the conceptual framework proposed by Andersen et al. [2]

In conclusion, observational comparisons between ECPR and CCPR face structural limitations that statistical adjustment alone cannot fully address. At prolonged low-flow durations, few clinically comparable CCPR patients remain, limiting causal inference. We propose a prioritized path forward. First, as a readily achievable step, registries could improve measurement by recording timestamps for the decision to proceed with ECPR, the start of cannulation, the establishment of ECMO flow, and termination of resuscitation, using standardized definitions, to allow accurate definition of time zero. Second, although more resource-intensive, a multicenter prospective observational study dedicated to collecting time-varying data would make it possible to quantify these biases directly. Finally, as the most challenging option, protocolized minimum CCPR durations in ECPR-eligible patients would restore comparators at long low-flow durations, but would require strong clinical and ethical justification. Until such data are available, these time-related biases should be explicitly acknowledged and assessed before causal conclusions are drawn from observational studies.

## Data Availability

No datasets were generated or analysed during the current study.

## Abbreviations

OHCA: Out-of-hospital cardiac arrest:
ECPR: Extracorporeal cardiopulmonary resuscitation
CCPR: Conventional cardiopulmonary resuscitation
ROSC: Return of spontaneous circulation
